# Predicting Temporal Gait Kinematics: Anthropometric Characteristics and Global Running Pattern Matter

**DOI:** 10.3389/fphys.2020.625557

**Published:** 2021-01-08

**Authors:** Aurélien Patoz, Thibault Lussiana, Cyrille Gindre, Laurent Mourot

**Affiliations:** ^1^Institute of Sport Sciences, University of Lausanne, Lausanne, Switzerland; ^2^Research and Development Department, Volodalen Swiss Sport Lab, Aigle, Switzerland; ^3^Research and Development Department, Volodalen, Chavéria, France; ^4^Research Unit EA3920 Prognostic Markers and Regulatory Factors of Cardiovascular Diseases and Exercise Performance, Health, Innovation Platform, University Bourgogne Franche-Comté, Besançon, France; ^5^Division for Physical Education, Tomsk Polytechnic University, Tomsk, Russia

**Keywords:** biomechanics, running, running speed, stride frequency, duty factor, predictive equation

## Abstract

Equations predicting stride frequency (SF) and duty factor (DF) solely based on running speed have been proposed. However, for a given speed, kinematics vary depending on the global running pattern (GRP), i.e., the overall individual movement while running, which depends on the vertical oscillation of the head, antero-posterior motion of the elbows, vertical pelvis position at ground contact, antero-posterior foot position at ground contact, and strike pattern. Hence, we first verified the validity of the aforementioned equations while accounting for GRP. Kinematics during three 50-m runs on a track (*n* = 20) were used with curve fitting and linear mixed effects models. The percentage of explained variance was increased by ≥133% for DF when taking into account GRP. GRP was negatively related to DF (*p* = 0.004) but not to SF (*p* = 0.08), invalidating DF equation. Second, we assessed which parameters among anthropometric characteristics, sex, training volume, and GRP could relate to SF and DF in addition to speed, using kinematic data during five 30-s runs on a treadmill (*n* = 54). SF and DF linearly increased and quadratically decreased with speed (*p* < 0.001), respectively. However, on an individual level, SF was best described using a second-order polynomial equation. SF and DF showed a non-negligible percentage of variance explained by random effects (≥28%). Age and height were positively and negatively related to SF (*p* ≤ 0.05), respectively, while GRP was negatively related to DF (*p* < 0.001), making them key parameters to estimate SF and DF, respectively, in addition to speed.

## Introduction

Running is defined as a cyclic alternance of lower limb support and flight phases, where at most one limb is in contact with the ground. In other words, the proportion of time spent by one limb in contact with the ground during a running stride, i.e., the duty factor (DF), is under 50% (Minetti, 1998; Folland et al., 2017). The running speed is determined by the product of the stride length and stride frequency (SF). All together, these three parameters allow defining running locomotion, out of which DF and SF, which control the temporal aspects of the running stride, are often of major interest due to their supposed relationship with running efficiency and performance (Moore, 2016). Moreover, DF was shown to be a good descriptor of running gait types (Fihl and Moeslund, 2007). Several studies have shown that with increasing speed, SF increases (Weyand et al., 2000; Mercer et al., 2002; Nummela et al., 2007) while DF decreases (Lussiana et al., 2019). On this basis, Gray et al. (2019) proposed quadratic regression equations to predict SF and DF solely based on speed. Their findings demonstrated that speed explained considerable variance in SF (*R*^2^ = 90.3%) while other parameters seemed required to explain variance in DF (*R*^2^ = 65.2%).

The considerably good estimation of SF (and DF, to some extent) provided by the equations proposed by Gray et al. (2019) might partly be due to their very specific cohort (10 young, well-trained, and healthy male soccer players). Recently, Moissenet et al. (2019) observed that lower limb sagittal gait kinematics in walking locomotion was predicted by walking speed but also by age, BMI, and sex. Similarly, a machine learning approach identified different running gait strategies where most of the groups differed in age or sex (Hoerzer et al., 2015). Other researchers showed that matching participants by height, mass, and sex was necessary to observe biomechanical differences between healthy and injured runners (Grau et al., 2008). Therefore, the results obtained by Gray et al. (2019) might suffer from a lack of generalization as anthropometric characteristics and sex could impact individual’s SF and DF and therefore their predictions. Furthermore, knowing which of these anthropometric characteristics improve predicting DF and SF could also help to better predict running efficiency and performance (Boullosa et al., 2020).

More specifically, DF was found to be highly variable among runners, with values ranging between 25 and 40% (average across three speeds: 2.78, 3.06, and 3.33 m/s) in a cohort of 97 endurance runners (Folland et al., 2017). Such variability could be attributed to participants’ intrinsic parameters such as age or sex (Cavagna et al., 2008b; Chapman et al., 2012). Indeed, Cavagna et al. (2008b) observed a lower flight time (*t*_*f*_) for old than for young men and Chapman et al. (2012) reported shorter ground contact time (*t*_*c*_) and swing time (*t*_*s*_) for women than men elite runners. However, these differences between sex were largely negated by normalizing to standing height (Chapman et al., 2012), which makes anthropometric characteristics potential candidates to impact DF. The spontaneous running pattern adopted by individuals could also impact DF. Indeed, a shorter *t*_*c*_ and larger *t*_*f*_ were observed for aerial (AER) than terrestrial (TER) runners, where these groups were defined based on a subjective evaluation of the global running pattern (GRP), i.e., an overall evaluation while running, which takes into account the vertical oscillation of the head, antero-posterior motion of the elbows, vertical pelvis position at ground contact, antero-posterior foot position at ground contact, and strike pattern (Gindre et al., 2016; Lussiana et al., 2017b). Therefore, taking GRP into account as an additional parameter when predicting DF could potentially improve the quality of its prediction. Noteworthy, understanding GRP of individuals might also assist in individualizing their training programs (Gindre et al., 2016).

On the contrary, no significant difference in step frequency, i.e., the half of SF, was observed between TER and AER runners for a given speed (range: 3.33–5.00 m/s) (Gindre et al., 2016), indicating that GRP might not be related to SF. Nevertheless, researchers observed large inter-individual differences in the spontaneous choice of SF at a given speed (Hunter and Smith, 2007; van Oeveren et al., 2017). These differences could be due to participants’ intrinsic parameters such as age (Cavagna et al., 2008a,b), sex (Chapman et al., 2012), mass (van Oeveren et al., 2019), and leg length (Heglund and Taylor, 1988; Cavagna et al., 1991; Marsh et al., 2004; Srinivasan and Ruina, 2006; van Oeveren et al., 2019), where greater SF was associated with older individuals, female, lower mass, and shorter leg length. In addition, as SF could be modified by gait retraining (Lenhart et al., 2014); experience, performance, and training could also impact SF. Indeed, van Oeveren et al. (2019) observed that higher training frequency and duration were positively related to SF, but running experience, performance, and injury incidence had no impact. Interestingly, these authors also observed that on an individual level, SF as a function of speed relationship was best described with a second-order polynomial while SF increased linearly with speed on a group level (van Oeveren et al., 2019). This result contradicts the observation of Gray et al. (2019) but might be due to the smaller range of speed and heterogeneous participant characteristics involved in the study of van Oeveren et al. (2019).

Hence, the purpose of this study was twofold. First, using a cohort of participants depicting similar characteristics than the one of Gray et al. (2019), we verified the validity of the regression equations relating SF and DF to speed while accounting for GRP. Kinematic differences being observed when classifying runners based on their GRP, we hypothesized that DF regression equation should no longer be valid when using such classification. On the contrary, as no difference in step frequency was reported for such classification, we hypothesized that SF regression equation should still be valid. Second, we generalized the SF and DF predictions by using a broader cohort of runners. Indeed, we assessed, in addition to speed, which parameters among anthropometric characteristics [age, height, mass, body mass index (BMI), and leg length], sex, training volume, and GRP could be related to SF and DF. We hypothesized that anthropometric characteristics, sex, and training volume should be related to both SF and DF, with SF and DF being linearly and quadratically dependent to speed, respectively. In addition, we hypothesized that GRP should be related to DF but not to SF.

## Materials and Methods

### Participant Characteristics

We conducted a retrospective analysis of data from our laboratory. For this investigation, we first selected SF and DF data from a group of 20 young trained males practicing a running related sport (team sports and triathlon): age: 22.3 ± 2.1 years, height: 181 ± 6 cm, mass: 71.7 ± 6.2 kg, BMI: 21.9 ± 1.4 kg/m^2^, and weekly training hours: 10.3 ± 4.9 h, while running on an athletic track. This group is the largest group available in our database while running on a track and sharing the same characteristics as in the study of Gray et al. (2019). These specific data were part of a larger data collection (91 individuals) that was used to verify that subjectively classified AER and TER runners were in fact associated with distinct objectively measured biomechanical parameters (Gindre et al., 2016).

Second, SF and DF were assessed when running on a treadmill using data from a different cohort of 54 trained runners composed of 21 females (age: 31.8 ± 8.7 years, height: 162 ± 4 cm, mass: 52.1 ± 5.5 kg, BMI: 19.8 ± 1.6 kg/m^2^, leg length: 83.8 ± 2.8 cm, and weekly running distance: 50.0 ± 21.0 km) and 33 males (age: 31.5 ± 9.3 years, height = 175 ± 7 cm, mass: 66.4 ± 10.7 kg, BMI: 21.6 ± 2.3 kg/m^2^, leg length: 91.1 ± 4.8 cm, and weekly running distance: 52.6 ± 20.2 km). These specific data have been previously collected to investigate the kinematic and energetic values between runners with a high and low DF at typical endurance running speeds (Lussiana et al., 2019).

All participants voluntarily participated in the original investigations and previously gave written informed consent. For study inclusion of the original investigations, participants were required to be in good self-reported general health with no current or recent (<3 months) musculoskeletal injuries. As for the original investigation on the treadmill, participants were also required to meet a certain level of running performance, i.e., a road race finishing time of ≤50 min for 10 km, ≤1 h 50 min for 21.1 km or ≤3 h 50 min for 42.2 km in the last year. These same criteria have been used for the retrospective analysis of data. All participants were familiar with running on a treadmill as part of their usual training program and wore their habitual running shoes during testing. The institutional review board approved this study protocol prior to participant recruitment (ID RCB 2016-A00500-51), which adhered to the latest Declaration of Helsinki of the World Medical Association.

### Experimental Procedure

As for the session on the athletic track, each participant ran for 10 min as a warm-up at a self-selected speed (range: 2.5–3.5 m/s). Then, the participant performed three randomized 50-m running trials at 3.33, 4.17, and 5.00 m/s starting from a standing-still position. Running trials were interspersed by 2-min rest periods during which time the participant was allowed to walk. Speed was monitored using photoelectric cells (Racetime2, MicroGate, Timing, and Sport, Bolzano, Italy) placed at the 20- and 40-m marks of the 50-m trial. A running trial was accepted when its speed was within ±5% of the specified speed. Otherwise, it was disregarded and repeated after a 2-min rest period, which occurred in less than 20% of the trials and no more than twice per participant.

As for the session on the treadmill, after measuring the right leg length of the participant (from anterior superior iliac spine to medial malleolus), the participant ran for 5 min at a self-selected speed (range: 2.8–3.5 m/s) on a treadmill (h/p/cosmos mercury^®^, h/p/cosmos sports & medical gmbh, Nussdorf-Traunstein, Germany) as a warm-up. Then, retro-reflective markers were positioned on individuals (described in section “Data Collection and Analysis”) to assess temporal gait kinematics. As for each participant, first, a 5-s standing static trial using a standard anatomical position was recorded on the treadmill for calibration purposes. This was followed by five 30-s runs at 2.78, 3.33, 3.89, 4.44, and 5.00 m/s (with 1-min recovery periods on the treadmill between each run). Three-dimensional (3D) kinematic data were collected during the static trial and the last 15 s of the running trials.

### Subjective Assessment of the Global Running Pattern

During the warm-up run at self-selected speed (on the athletic track or treadmill), an expert running coach with 5 years of experience using the Volodalen^®^ scale focused on the overall movement of participants. The coach paid attention to five key elements: vertical oscillation of the head, antero-posterior motion of the elbows, vertical pelvis position at ground contact, antero-posterior foot position at ground contact, and strike pattern (Gindre et al., 2016; Lussiana et al., 2017a). Each element was scored from one to five, leading to a subjective score (V^®^score) that represents GRP of participants. This score ultimately allows the classification of participants in two different groups termed TER (V^®^score ≤ 15) and AER (V^®^score > 15). The intra- and inter-rater reliability of this method has been shown recently (Patoz et al., 2019a). This method has been recognized as a reliable evaluation of GRP, being strongly correlated to the measurements of biomechanical parameters (Lussiana et al., 2017b).

### Data Collection and Analysis

As for the session on the athletic track, a 20-m optical measurement system (Optojump Next^®^, MicroGate Timing and Sport, Bolzano, Italy) sampling at 1000 Hz was used to record *t*_*c*_ and *t*_*f*_ between the 20- and 40-m marks of the 50-m trial. As no distinction between right and left limbs were made, *t*_*s*_ was, on average, given by *t*_*s*_ = *t*_*c*_ + 2*t*_*f*_.

As for the session on the treadmill, 3D kinematic data were collected at 200 Hz and a resolution of 1.3 megapixels using seven infrared Oqus cameras (five Oqus 300+, one Oqus 310+, and one Oqus 311+) and Qualisys Track Manager software version 2.1.1 build 2902 together with the Project Automation Framework Running package version 4.4 (Qualisys AB, Göteborg, Sweden). Four retro-reflective markers of 12 mm diameter were used for static and running trials and were affixed to the shoes of individuals over anatomical landmarks (dorsal aspect of the second metatarsal head and aspect of the Achilles tendon insertion on the calcaneus) using double-sided tape following standard guidelines from the Project Automation Framework Running package (Tranberg et al., 2011) and as already reported elsewhere (Lussiana et al., 2019). The 3D marker data were exported in .c3d format and processed in Visual3D Professional software version 5.02.25 (C-Motion Inc., Germantown, MD, United States). More explicitly, the 3D marker data were interpolated using a third-order polynomial least-square fit algorithm (using three frames of data before and after the “gap” to calculate the coefficients of the polynomial), allowing a maximum of 20 frames for gap filling, and subsequently low-pass filtered at 20 Hz using a fourth-order Butterworth filter. Running events were derived from the trajectories of the 3D marker and using similar procedures to those previously reported in the literature (Maiwald et al., 2009; Lussiana et al., 2019). More explicitly, a mid-foot landmark was generated midway between the heel and toe markers. Footstrike was defined as the instance when the mid-foot landmark reached a local minimal vertical velocity prior to it reaching a peak vertical velocity reflecting the start of swing. Toe-off was defined as the instance when the toe marker attained a peak vertical acceleration before reaching a 7-cm vertical position. *t*_*s*_ and *t*_*c*_ were defined as the time from toe-off to footstrike and from footstrike to toe-off of the same foot, respectively. All events were verified to ensure correct identification and were manually adjusted when required.

Based on *t*_*c*_ and *t*_*s*_ values, DF was calculated as $\text{DF}=\frac{t_{c}}{t_{c}+t_{s}}$(Minetti, 1998) and SF was given by $\text{SF}=\frac{1}{t_{c}+t_{s}}.$ *t*_*c*_ and *t*_*s*_ represent the average between right and left values. Gray et al. (2019) proposed to describe these two parameters using the following quadratic regression equations (Eqs. 1 and 2)

(1) $$\text{SF}=0.026\,s^{2}-0.111\,s+1.398\left(R_{\text{adj}}^{2}=90.2\%,\text{SE}=0.21\mathrm{Hz}\right)$$

(2) $$\text{DF}=0.4s^{2}-6.1s+50\left(R_{\text{adj}}^{2}=64.9\%,\text{SE}=7.4\%\right)$$

where *s* represents speed (in m/s) and SE denotes the standard error of the fit. In this study, a similar curve fitting procedure based on a second-order polynomial was applied to SF and DF obtained from the session on the athletic track with and without the subgrouping of participants. SE and $R_{\text{adj}}^{2}$ were computed to assess the quality of the fit. Data analysis was performed using Python (version 3.7.4, Python Software Foundation, available at http://www.python.org).

### Statistical Analysis

Descriptive statistics are presented using mean ± standard deviation (SD) unless otherwise indicated. The normality of the data and homogeneity of variances were verified using Kolmogorov–Smirnov and Levene’s test, respectively. Bland–Altman plots (Bland and Altman, 1995; Atkinson and Nevill, 1998) were constructed to examine the presence of systematic and proportional biases between calculated SF and DF from the data obtained during the session on the athletic track and “theoretical” ones computed using Eqs. 1 and 2 provided by Gray et al. (2019). Systematic bias was also identified by a significant difference obtained from a paired Student’s *t*-test. After confirming visually that no correlation was present among the residuals, the proportional bias (heteroscedasticity) was identified by a significant slope of the regression line. Participant characteristics between groups were compared using unpaired Student’s *t*-tests. The effect of speed on SF and DF was evaluated using a linear mixed effects model fitted by restricted maximum likelihood. The within-subject nature was controlled for by including random effects for participant (subject-specific effects). As for the session on the athletic track, the fixed effects included speed and GRP (continuous variables) using the following model (Eq. 3):

(3) $$Y=b_{0}+b_{1}\,s+b_{2}\,s^{2}+c_{1}\,\text{GRP}+d_{1}\,\left(s\times \text{GRP}\right)+\left\{\text{intercept}+s|\text{part}\,\_\,\text{id}\right\}$$

where *Y* represents SF or DF, *s* is speed, the *b*_*i*_’s, *c*_1_, and *d*_1_ are the regression coefficients, (*s* × GRP) is the interaction term, and the term within curly brackets denotes the random effects’ term: a participant identifier (part_id) was used as a random effect variable to account for individual differences in the intercept and linear *s* terms. Of note, a random effect in the quadratic *s*^2^ term could not be used due to the fact that three random terms applied on 20 participants require >60 observations but 60 observations were made.

As for the session on the treadmill, the fixed effects included speed (continuous variable), sex (categorical variable), and variables among age, height, mass, BMI, leg length, weekly running distance, and GRP that correlated to less than 0.7 to prevent collinearity (van Oeveren et al., 2019). Correlation was determined using Pearson correlation coefficient (*r*), calculated between each pair of previously mentioned variables. Very high, high, moderate, low, and negligible correlations are given by the following *r* values, respectively: 0.90–1.00, 0.70–0.90, 0.50–0.70, 0.30–0.50, and 0.00–0.30 (Hinkle et al., 2003). Based on correlation coefficients; age, height, BMI, weekly running distance, and GRP (continuous variables) were used as fixed effects on the model, which was as follows (Eq. 4):

(4) $$Y=b_{0}+b_{1}s+b_{2}s^{2}+\sum_{i=1}^{5}\left[c_{i}v_{i}+d_{i}\left(s\times v_{i}\right)\right]+0.5[c_{6}\text{sex}+d_{6}\left(s\times \text{sex}\right)]+\{\text{intercept}+s+s^{2}|\text{part}\_\text{id}\}$$

where *Y* represents SF or DF, *s* is speed, the *b*_*i*_’s, *c*_*i*_’s, and *d*_*i*_’s are the regression coefficients, the sex is 1 for female and -1 for male, the *v*_*i*_’s are as follows: *v*_1_: age, *v*_2_: height, *v_3_:* BMI, *v*_4_: weekly running distance, and *v*_5_: GRP, (*s* × *v*_*i*_) and (*s* × sex) are the interaction terms, and the term within curly brackets denotes the random effects’ term (individual differences in intercept, linear *s*, and quadratic *s*^2^ terms were taken into account).

Continuous independent variables were centered around the mean. The variance explained by the fixed effects over the total expected variance of the dependent variable was given by $R_{\text{marginal}}^{2}$ while $R_{\text{conditional}}^{2}$ represented the variance explained by the fixed and random effects together over the total variance of the dependent variable (Johnson, 2014). Intra-class correlation coefficients (ICCs) of the random effects were computed as the ratio of the variance of the random coefficient divided by the sum of itself and the residual variance. On the basis of commonly used thresholds, poor, moderate, good, and excellent ICCs are given by ICC values <0.5, 0.5–0.75, 0.75–0.90, and ≥0.90, respectively (Koo and Li, 2016). Likelihood ratio tests were employed to assess the relevance of using several random effects. Statistical analysis was performed using Jamovi (version 1.2.17, Computer Software, retrieved from https://www.jamovi.org) with a level of significance set at *p* ≤ 0.05.

## Results

### Session on the Athletic Track

**FIGURE 1:**
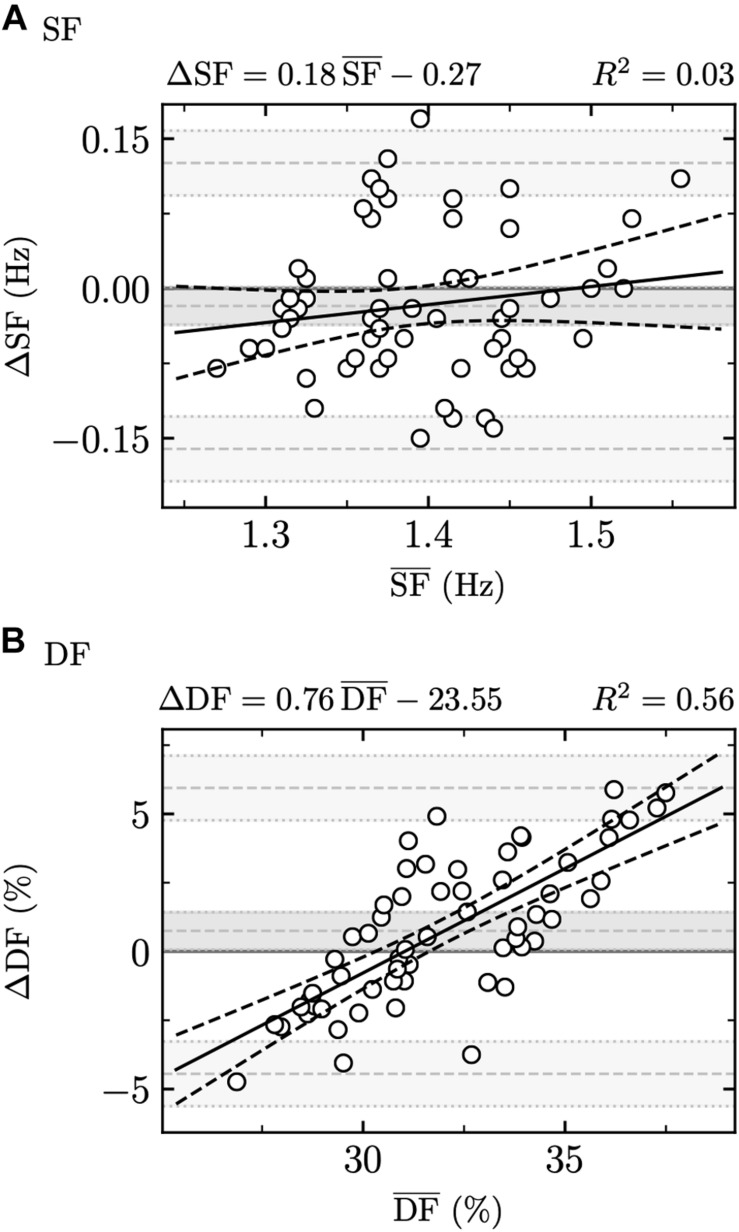
Bland–Altman plots that compare calculated and theoretical **(A)** stride frequency (SF) and **(B)** duty factor (DF). Calculated values were obtained during the session on the athletic track while theoretical ones were computed using Eqs. 1 and 2 proposed by Gray et al. (2019). A significant bias is reported for DF (the horizontal line at ΔDF = 0 is not within the 95% confidence interval given by the dark gray shaded area) but not for SF.

The Bland–Altman plots comparing calculated SF and DF to theoretical ones are depicted in Figure 1. The bias ± random error (1.96 SD) of SF and DF were −0.02 ± 0.14 Hz and 0.75 ± 5.20%, respectively. Significant systematic (*p* = 0.03) and proportional (slope ± SE = 0.76 ± 0.09, *p* < 0.001) biases were obtained for DF but not for SF (*p* ≥ 0.07, slope ± SE = 0.18 ± 0.14).

**FIGURE 2:**
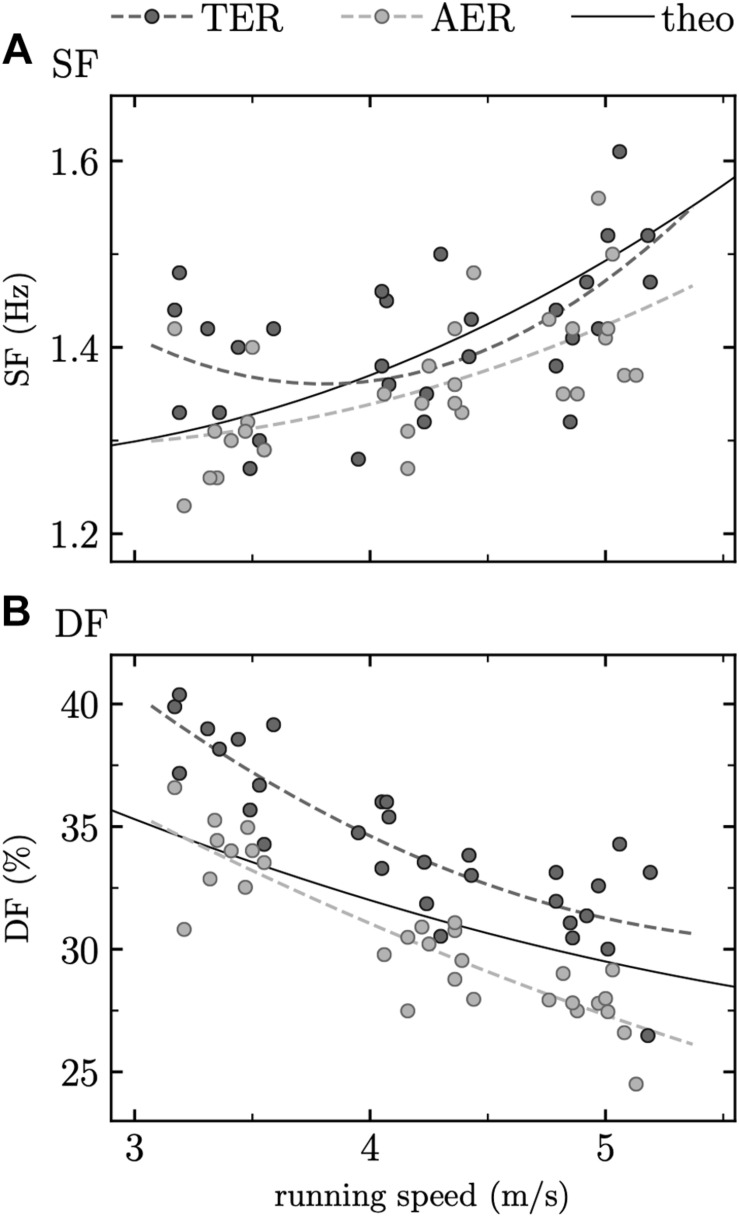
Calculated (circle) and fitted (dashed line) **(A)** stride frequency (SF) and **(B)** duty factor (DF) for terrestrial (TER) and aerial (AER) runners together with regression equations (theo) proposed by Gray et al. (2019) (plain line, SF: Eq. 1, and DF: Eq. 2) for which no group distinction was made.

Second-order polynomial curve fitting applied to the data of the 20 participants pooled together led to the following relationships (Eqs. 5 and 6):

(5) $$\text{SF}=0.052\,s^{2}-0.372\,s+2.004\left(R_{\text{adj}}^{2}=30.0\%,\text{SE}=0.07\text{Hz}\right)$$

(6) $$\text{DF}=0.90s^{2}-11.6s+64.9\left(R_{\text{adj}}^{2}=54.8\%,\text{SE}=2.5\%\right)$$

where *s* represents speed. Then, the 20 participants were classified based on their GRP, which led to 10 participants in TER (V^®^score: 12.7 ± 2.5, age: 22.6 ± 2.2 years, height: 183 ± 5 cm, mass: 73.5 ± 4.8 kg, BMI: 21.9 ± 1.4 kg/m^2^, and weekly training hours: 10.0 ± 3.5 h) and AER (V^®^score: 19.3 ± 2.5, age: 22.0 ± 2.1 years, height: 179 ± 6 cm, mass: 69.8 ± 7.0 kg, BMI: 21.8 ± 1.6 kg/m^2^, and weekly training hours: 10.6 ± 6.3 h) groups. No significant differences were revealed for participant characteristics (age, height, mass, BMI, and weekly training hours) between groups (*p* ≥ 0.08). The corresponding regression equations obtained using quadratic curve fitting are given by Eqs. 7–10.

(7) $${\text{SF}}_{\text{TER}}=0.077\,s^{2}-0.589\,s+2.481\left(R_{\text{adj}}^{2}=31.2\%,\text{SE}=0.07\text{Hz}\right)$$

(8) $${\text{SF}}_{\text{AER}}=0.022\,s^{2}-0.112\,s+1.438\left(R_{\text{adj}}^{2}=36.8\%,\text{SE}=0.06\text{Hz}\right)$$

(9) $${\text{DF}}_{\text{TER}}=1.22\,s^{2}-14.33\,s+72.42\left(R_{\text{adj}}^{2}=72.7\%,\text{SE}=1.67\%\right)$$

(10) $${\text{DF}}_{\text{AER}}=0.39\,s^{2}-7.21\,s+53.70\left(R_{\text{adj}}^{2}=78.9\%,\text{SE}=1.33\%\right)$$

where *s* is speed. These equations are also represented in Figure 2 together with theoretical models proposed by Gray et al. (2019).

The linear mixed effects model reported that both speed and the square of speed were significantly related to SF and DF (*p* ≤ 0.002; Table 1). The regression coefficients imply that for each 1 m/s increase in speed, SF is increased by 0.064 Hz, and for each 1 m^2^/s^2^ increase in the square of speed, SF is increased by 0.027 Hz (Table 1). The DF model indicated that for each 1 m/s increase in speed, DF is decreased by 4.01% while for each 1 m^2^/s^2^ increase in the square of speed, DF is increased by 1.12% (Table 1). In addition, GRP was negatively related to DF (*p* < 0.004; Table 1), with a 1 point increase in V^®^score leading to a decrease in DF of 0.36% (Table 1). The model with random effects explained almost all variance in the data for both SF and DF ($R_{\text{conditional}}^{2}\geq$95.4% versus $R_{\text{marginal}}^{2}$ ≤ 69.3%; Table 1). The ICCs of the random effects were excellent for the intercept and moderate for speed, for both SF and DF models (Table 1). The significant likelihood ratio tests obtained for both SF and DF models allowed us to conclude that using {intercept + *s* | part_id} random effect was better than using {intercept | part_id} random effect (*p* ≤ 0.03; Table 1).

**TABLE 1:** Regression coefficients [estimate ± standard error (SE) together with 95% confidence interval (CI)] as defined in Eq. 9, percentage of variance explained, fixed effects, and random effects [variance, intra-class correlation coefficient (ICC), and likelihood ratio test (LRT)] when assessing the effect of speed (s) and global running pattern (GRP) on stride frequency (SF) and duty factor (DF) obtained during the session on the athletic track using a linear mixed effects model.

	SF	DF

Regression coefficients	Estimate ± SE (95% CI)	Estimate ± SE (95% CI)
*b*_0_ (intercept)	1.372 ± 0.014 (1.345, 1.400)	31.91 ± 0.45 (31.03, 32.78)
*b*_1_ (*s*)	0.064 ± 0.007 (0.050, 0.077)	−4.01 ± 0.22 (−4.44, −3.57)
*b*_2_ (*s*^2^)	0.027 ± 0.007 (0.012, 0.042)	1.12 ± 0.24 (0.64, 1.59)
*c*_1_ (GRP)	−0.006 ± 0.004 (−0.013, 0.000)	−0.36 ± 0.11 (−0.57, −0.14)
*d*_1_ (*s* × GRP)	0.002 ± 0.002 (−0.002, 0.005)	0.01 ± 0.05 (−0.09, 0.12)

**Variance explained**	**%**	**%**

$R_{\text{marginal}}^{2}$	35.9	69.3
$R_{\text{conditional}}^{2}$	95.4	97.7

**Fixed effects**	***p***	***p***

Intercept	**<0.001**	**<0.001**
*s*	**<0.001**	**<0.001**
*s*^2^	**0.002**	**<0.001**
GRP	0.08	**0.004**
*s* × GRP interaction	0.38	0.83
**Random effects**	–	–
Variance for intercept	4E-3	3.77
Variance for *s*	6E-4	0.71
ICC for intercept	0.92	0.92
ICC for *s*	0.69	0.69
LRT for *s* in {intercept + *s* | part_id} (*p*)	**0.03**	**0.02**

*Statistical significances (*p* ≤ 0.05) are indicated in bold.*

### Session on the Treadmill

The 54 participants were classified based on their GRP, which led to 26 participants in TER (V^®^score: 11.4 ± 2.4, age: 31.7 ± 8.9 years, height: 168 ± 9 cm, mass: 58.6 ± 9.3 kg, BMI: 20.7 ± 1.9 kg/m^2^, leg length: 87.3 ± 5.8 cm, weekly running distance: 53.7 ± 21.1 km, 10 males, and 16 females) and 28 in AER (V^®^score: 19.3 ± 2.6, age: 31.5 ± 9.2 years, height: 172 ± 8 cm, mass: 62.9 ± 12.9 kg, BMI: 21.0 ± 2.5 kg/m^2^, leg length: 89.1 ± 5.1 cm, and weekly running distance: 49.6 ± 19.8 km, 23 males, and 5 females) groups. No significant differences were revealed for participant characteristics (age, height, mass, BMI, leg length, and weekly running distance) between groups (*p* ≥ 0.10).

High and very high correlations were obtained between height and mass, height and leg length, BMI and mass, and mass and leg length (*r* ≥ 0.81, *p* < 0.001; Table 2); leg length and mass were not included in the linear mixed effects model.

**TABLE 2:** Correlation matrix providing the Pearson’s correlation coefficients (*r*) for each pair of variables among age, height, mass, BMI, leg length, weekly running distance, and global running pattern (GRP), together with their corresponding statistical significance (*p* ≤ 0.05), indicated in bold.

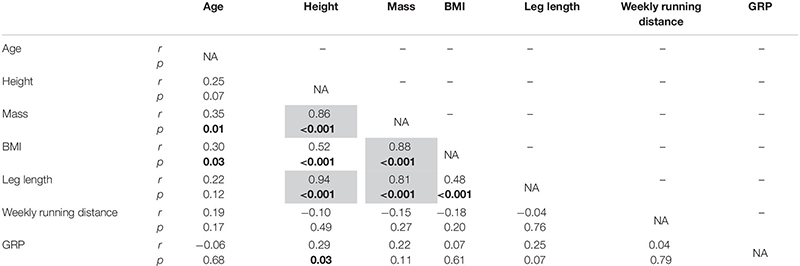

*Gray shaded boxes denote correlation coefficients above *r* = 0.7 and were not analyzed together in the linear mixed effects model to prevent collinearity. BMI, body mass index and NA, not applicable.*

The linear mixed effects model reported that both speed and age were positively related to SF (*p* ≤ 0.05; Table 3) while height was negatively related to SF (*p* = 0.01; Table 3). The regression coefficients imply that for each 1 m/s increase in speed, SF is increased by 0.094 Hz; for a 1-year increase in age, SF is increased by 0.003 Hz, and being 10 cm taller corresponds to a decrease of SF by 0.054 Hz (Table 3). Both speed and the square of speed as well as GRP and speed × GRP interaction were significantly related to DF (*p* ≤ 0.03; Table 3). The DF model indicated that for each 1 m/s increase in speed, DF is decreased by 3.77%, while for each 1 m^2^/s^2^ increase in the square of speed, DF is increased 1.05% (Table 3). In addition, for a 1 point increase in V^®^score, DF is decreased by 0.40% and an individual with a V^®^score of 19 running at 3 m/s would have a DF 2.29% higher than an individual with a V^®^score of 12, while at 5 m/s, the difference would be 3.82% (Table 3). The model with random effects explained almost all variance in the data for both SF and DF ($R_{\text{conditional}}^{2}$ ≥ 96.4% versus $R_{\text{marginal}}^{2}$ ≤ 66.5%; Table 3). The ICCs of the random effects for the SF model were excellent for the intercept, good for speed, and moderate for the square of speed (Table 3). As for the DF model, The ICCs of the random effects were good for the intercept and moderate for both speed and the square of speed (Table 3). The significant likelihood ratio tests obtained for both SF and DF models allowed us to conclude that using {intercept + *s*+ *s*^2^ | part_id} random effect was better than using {intercept | part_id} random effect (*p* < 0.001; Table 3).

**TABLE 3:** Regression coefficients [estimate ± standard error (SE) together with 95% confidence interval (CI)] as defined in Eq. 10, percentage of variance explained, fixed effects, and random effects [variance, intra-class correlation coefficient (ICC), and likelihood ratio test (LRT)] when assessing the effect of speed (s), age, height, body mass index (BMI), weekly running distance, global running pattern (GRP), and sex on stride frequency (SF) and duty factor (DF) obtained during the session on the treadmill using a linear mixed effects model.

	SF	DF

Regression coefficients	Estimate ± SE (95% CI)	Estimate ± SE (95% CI)
*b*_0_ (intercept)	1.509 ± 0.012 (1.486, 1.532)	33.15 ± 0.35 (32.47, 33.83)
*b*_1_ (*s*)	0.094 ± 0.005 (0.085, 0.103)	−3.77 ± 0.20 (−4.16, −3.37)
*b*_2_ (*s*^2^)	0.006 ± 0.003 (−5E-4, 0.012)	1.05 ± 0.16 (0.73, 1.37)
*c*_1_ (age)	0.003 ± 0.001 (1E-4, 0.006)	0.05 ± 0.04 (−0.02, 0.13)
*c*_2_ (height)	−0.549 ± 0.198 (−0.936, −0.162)	6.58 ± 5.47 (−4.14, 17.31)
*c*_3_ (BMI)	−0.009 ± 0.006 (−0.021, 0.002)	−0.13 ± 0.16 (−0.45, 0.19)
*c*_4_ (weekly running distance)	4E-5 ± 6E-4 (−0.001, 0.001)	−0.02 ± 0.02 (−0.05, 0.01)
*c*_5_ (GRP)	−0.004+0.003 (−0.009, 0.001)	−0.40 ± 0.08 (−0.55, −0.26)
*c*_6_ (sex)	−0.016 ± 0.038 (−0.090, 0.058)	−0.57 ± 1.04 (−2.61, 1.48)
*d*_1_ (*s* × age)	4E-4 ± 5E-4 (−7E-4, 0.001)	0.01 ± 0.03 (−0.04, 0.06)
*d*_2_ (*s* × height)	−0.087 ± 0.078 (−0.240, 0.066)	−1.30 ± 3.50 (−8.16, 5.55)
*d*_3_ (*s* × BMI)	0.001 ± 0.002 (−0.003, 0.006)	0.01 ± 0.10 (−0.19, 0.22)
*d*_4_ (*s* × weekly running distance)	−4E-5 ± 2E-4 (−5E-4, 4E-4)	9E-4 ± 0.01 (−0.02, 0.02)
*d*_5_ (*s* × GRP)	0.001 ± 0.001 (−0.003, 0.001)	0.11 ± 0.05 (0.02, 0.20)
*d*_6_ (*s* × sex)	0.011 ± 0.015 (−0.018, 0.041)	0.67 ± 0.67 (−0.63, 1.98)

**Variance explained**	**%**	**%**

$R_{\text{marginal}}^{2}$	56.9	66.5
$R_{\text{conditional}}^{2}$	98.3	96.4

**Fixed effects**	***p***	***p***

Intercept	**<0.001**	**<0.001**
*s*	**<0.001**	**<0.001**
*s*^2^	0.08	**<0.001**
Age	**0.05**	0.19
Height	**0.01**	0.24
BMI	0.11	0.42
Weekly running distance	0.95	0.26
GRP	0.16	**<0.001**
Sex	0.67	0.59
*s* × age	0.46	0.66
*s* × height	0.27	0.71
*s* × BMI	0.59	0.92
*s* × weekly running distance	0.86	0.93
*s* × GRP interaction	0.36	**0.03**
*s* × sex	0.44	0.32
**Random effects**	–	–
**Fixed effects**	***p***	***p***
Variance for intercept	6E-3	5.47
Variance for *s*	1E-3	1.66
Variance for *s*^2^	4E-4	0.93
ICC for intercept	0.96	0.89
ICC for *s*	0.78	0.71
ICC for *s*^2^	0.57	0.57
LRT for *s* in {intercept + *s*+ *s*^2^ | part_id} (*p*)	**<0.001**	**<0.001**
LRT for *s*^2^ in {intercept + *s*+ *s*^2^ | part_id} (*p*)	**<0.001**	**<0.001**

*Statistical significances (*p* ≤ 0.05) are indicated in bold.*

## Discussion

In accordance with our first hypothesis, the DF regression equation proposed by Gray et al. (2019) was not applicable when GRP was taken into account, while such parameter had no impact on SF regression equation. We also found that GRP was negatively related to DF but not to SF, supporting these observations. Our second hypothesis, however, was partly refuted. Indeed, even if a linear dependence with speed was obtained for SF when using a broader cohort of runners, only age and height (among age, height, BMI, weekly running distance, sex, and GRP) were positively and negatively related to SF, respectively. As for DF, though a quadratic dependence with speed was obtained, only GRP was negatively related to DF. In addition, a positive relation between speed × GRP interaction and DF was found.

The present findings showed no systematic or proportional biases when comparing SF values obtained from athletic track measurements to theoretical values using Eq. 1 proposed by Gray et al. (2019) (Figure 1) and a similar cohort in terms of participant characteristics. This result demonstrates that the quadratic relationship between SF and speed proposed by Gray et al. (2019) seems to be consistent for such specific cohort. However, a much smaller $R_{\text{adj}}^{2}$ was obtained here than by these authors (90.2 versus 30.0%), though associated with a lower SE (0.21 versus 0.07 Hz). The reason of the smaller SE might come from the smaller number of observations made here (20 participants and 3 speeds versus 10 participants, 6 speeds, and 2 trials per participant), leading to a smaller residual sum of squares error (SSR), and thus a smaller SE. The smaller $R_{\text{adj}}^{2}$ obtained here might be due to the smaller range of speed employed (3.33–5.00 m/s) and corresponding smaller range of SF observed for these speeds than in Gray et al. (2019). Indeed, this might have led to a smaller total sum of squares error (SST), and the small SSR and SST obtained here gave a SSR/SST ratio larger than that in Gray et al. (2019) and thus a smaller $R_{\text{adj}}^{2}.$ Moreover, the smaller speed range used in this study gave more weights to the lower end of the speed interval (3.33 m/s), leading to more pronounced differences with respect to the results of Gray et al. (2019) on the low-speed side than on the high-speed side. SF regression equations for TER and AER runners (Eqs. 7 and 8; $R_{\text{adj}}^{2}$ ≤ 36.8%; SE ≥ 0.06 Hz), i.e., taking into account individual GRP to estimate SF, were not explaining more variance or having a smaller SE than the regression equation without subgrouping of participants (Eq. 5; $R_{\text{adj}}^{2}$ = 30.0%; SE = 0.07 Hz). In addition, the linear mixed effects model depicted no effect of GRP on SF (Table 1). These results agree with the fact that TER and AER runners were shown to share the same step frequency (Gindre et al., 2016). Nevertheless, large inter-individual differences in the spontaneous choice of SF was depicted by the fact that the model with random effects explained more and almost all variance in the data than a model with only fixed effects ($R_{\text{conditional}}^{2}$ = 95.4% versus $R_{\text{marginal}}^{2}$ ≤ 35.9%; Table 1) and by the fact that the ICCs of the random effects were excellent for the intercept and moderate for speed (Table 1). Therefore, as the cohort shared the same participant characteristics, such SF variability on an individual level might be related not only to participants’ intrinsic parameters such as age (Cavagna et al., 2008b, a), sex (Chapman et al., 2012), mass (van Oeveren et al., 2019), leg length (Heglund and Taylor, 1988; Cavagna et al., 1991; Marsh et al., 2004; Srinivasan and Ruina, 2006; van Oeveren et al., 2019), and training frequency and duration (Heglund and Taylor, 1988; Cavagna et al., 1991; Marsh et al., 2004; Srinivasan and Ruina, 2006; van Oeveren et al., 2019), but also to the subconscious fine-tuning of running biomechanics referred to as self-optimization (Moore, 2016). Nonetheless, the large random effects observed here could also be due to the small range of tested speeds (3.33–5.00 m/s); thus, applying the same linear mixed effects model to a dataset containing a larger speed range such as the one of Gray et al. (2019) would prove to be useful to validate this assumption. The range of speed we selected here is, however, in line with most of the running speeds faced by endurance runners and could be considered as representative of this physical activity.

The comparison between DF obtained from athletic track measurements and Eq. 2 proposed by Gray et al. (2019) depicted a slightly smaller $R_{\text{adj}}^{2}$ and a lower SE in this study than in Gray et al. (2019) ($R_{\text{adj}}^{2}$: 64.9 versus 54.8%; SE: 7.4 versus 2.5%). The smaller difference in $R_{\text{adj}}^{2}$ obtained for DF than for SF between measured and theoretical values might be due to the larger variations of DF than SF values within the speed range employed (3.33–5.00 m/s), leading to a larger SST and thus smaller SSR/SST ratio for DF than for SF. However, systematic and proportional biases were obtained for DF (Figure 1) despite the similar participant characteristics. As pointed out by Gray et al. (2019), due to the fact that a $R_{\text{adj}}^{2}$ of 64.9% was obtained for DF regression, DF seemed to rely on factors other than speed. Therefore, we took into account GRP in the DF estimation, which is in line with the fact that a shorter *t*_*c*_ and larger *t*_*f*_ were observed for AER than TER runners (Gindre et al., 2016; Lussiana et al., 2017b) and that classifying runners in two groups based on their DF led to kinematic differences of their running gait (Lussiana et al., 2019) as well as similar subgroupings of runners between classifications based on GRP and DF (Patoz et al., 2019b). DF regression equations for AER and TER runners led to a reduction of SE by ≥147% and an improvement of $R_{\text{adj}}^{2}$ of ≥133%. Therefore, including such classification increased the quality of the fit and percentage of explained variance. This was reinforced by the negative relation between GRP and DF depicted in the linear mixed effects model, in addition to the negative and positive effects observed for speed and the square of speed, respectively (Table 1). Similarly to SF, inter-individual differences in the spontaneous choice of DF was depicted by a greater $R_{\text{conditional}}^{2}$ = 97.7% than $R_{\text{marginal}}^{2}$=69.3% (Table 1), and by the ICCs of the random effects which were excellent for the intercept and moderate for speed (Table 1). Therefore, DF variability on an individual level might also be related to self-optimization, as this was shown to be present in *t*_*c*_ (Moore, 2016; Moore et al., 2019), but again, using the same linear mixed effects model on a dataset with a larger speed range would be needed to validate this assumption.

As for treadmill data, the high and very high correlations obtained between height and mass, height and leg length, BMI and mass, and mass and leg length (*r* ≥ 0.81; Table 2) are in agreement with previous observations (van Oeveren et al., 2019). Indeed, these authors also obtained correlation coefficients above 0.7 except between leg length and mass (but quite close; *r* = 0.66). The correlation coefficient between leg length and mass obtained here was the smallest one among the ones being above 0.7, which is consistent with the observations of van Oeveren et al. (2019). The presence of such correlations forced us to not use leg length and mass in the linear mixed effects model, so that any collinearity be avoided.

Stride frequency was linearly related to speed at a group level (Table 3), as already observed (van Oeveren et al., 2019). On an individual level, variations in SF were relatively large, as depicted by the improvement of explained variance when using random effects ($R_{\text{conditional}}^{2}$ = 98.3% versus $R_{\text{marginal}}^{2}$ = 56.9%; Table 3) and the moderate to excellent ICCs of the random effects (ICC ≥ 0.57; Table 3). Moreover, the significant likelihood ratio tests (*p* < 0.001; Table 3) demonstrated that SF as a function of speed relationship was best described by a second-order polynomial, which confirms results from previous studies (Weyand et al., 2000; Mercer et al., 2002; Nummela et al., 2007; van Oeveren et al., 2019). The small range of speed (2.78–5.00 m/s) might partly explain why the quadratic term in the linear mixed effects model was not significant. Indeed, the quadratic behavior starts to be visible at a speed above 5 m/s on a group level [e.g., see Figure 3 in van Oeveren et al. (2019)]; therefore, the small speed range employed here might have smoothed out the quadratic behavior observed on an individual level to a linear behavior on a group level. Another part of the explanation might be that individuals may preferentially rely either on an increase of SF or on stride length to increase speed, leading to a different sign for the quadratic coefficient, thus canceling each other at a group level, as already pointed out (van Oeveren et al., 2019). Taller runners had lower SF (Table 3). As leg length was very highly correlated to height (Table 2), runners with longer legs presumably had lower SF, which is in agreement with previous findings (van Oeveren et al., 2019). Older runners had higher SF (Table 3), suggesting that less horizontal propulsion (longer stride length) is achievable with increasing age. A similar observation was made by Cavagna et al. (2008b) when studying two groups of runners with larger age difference and was attributed to a reduction of muscle mass with age. No effect of GRP on SF was observed (Table 3), suggesting that GRP does not impact SF, as already observed for the athletic track session and between AER and TER runners (Gindre et al., 2016). The weekly running distance was not significantly related to SF, meaning that training volume is not a determinant factor of SF. This result is opposed to the observation of van Oeveren et al. (2019) and possibly explained by the fact that the weekly running distance was not broad enough to reflect a relation with SF.

Duty factor was quadratically related to speed (Table 3), which is in line with the results obtained from the athletic track measurements and by Gray et al. (2019). On an individual level, DF had smaller inter-individual variations than SF ($R_{\text{conditional}}^{2}$=96.4% versus $R_{\text{marginal}}^{2}$ = 66.5%; Table 3) but still non-negligible, as depicted by the moderate and high ICCs of the random effects (ICC ≥ 0.57; Table 3). GRP was negatively related to DF, which is in agreement with TER runners spending more time in contact with the ground but less time in the air than AER runners (Gindre et al., 2016; Lussiana et al., 2017b). A positive speed × GRP interaction was observed (Table 3), suggesting that a greater decline in DF with the increase of speed is observed for a lower V^®^score. TER runners having a larger DF than AER suggests that TER runners have a larger adaptation range of DF with increasing speed than AER runners. Moreover, increasing running speed leads to a running pattern that tends to be more aerial (more bouncy). This could suggest that faster runners, due to the fact that they train at higher running speeds, have a more aerial running pattern. This in agreement with the findings of da Rosa et al. (2019), which showed that high-performance runners had longer *t*_*f*_ than low-performance runners, and therefore, these high-performance runners demonstrated a better use of elastic components. Age and sex were not related to DF, meaning that the observations of a lower *t*_*f*_ for old than for young men (Cavagna et al., 2008b) and a shorter *t*_*c*_ for women than for men elite runners (Chapman et al., 2012) were not as strongly present here to be reflected in DF. These differences might be due to the fact that the old participants in Cavagna et al. (2008b) were much older than the participants of this study (oldest participant: 51 years) and that no elite runners were tested.

A few limitations to the present study exist. The number of speeds tested was limited for the athletic track session, and additional conditions (speeds, slopes, and types of ground) would be necessary to obtain universal equations. However, the main purpose being the generalization of SF and DF prediction equations (by using a broader cohort of runners and by taking into account anthropometric characteristics, sex, training volume, and GRP in addition to speed), the strength of these results was given by the large dataset employed in this part of the study. Nonetheless, treadmill instead of athletic track data were used to study the relation between participant characteristics and both temporal gait kinematic parameters because this was the largest dataset available to us. SF and DF models obtained from treadmill measurements might be applied to data recording on the athletic track due to the fact that spatiotemporal parameters between motorized treadmill and overground running are largely comparable (Van Hooren et al., 2020). However, it was also concluded that participants behaved differently when attempting to achieve faster running velocities overground than on a treadmill (Bailey et al., 2017). Therefore, further studies should focus on prediction equations of temporal gait kinematic parameters based on athletic track data. Then, these equations could be compared to the treadmill ones. In case of any differences, a mathematical relation could be constructed so that one could easily switch from treadmill to overground prediction by providing either treadmill or overground data. Also, the four linear mixed effects models did not explain more than 69.3% of the variance without random effects while the random effects explained more than 95.4% of the variance, leading to ≥28% of the variance that is unexplained inter-individual variation, meaning that parameters related to SF and DF might still be missing in the model. Further research including internal factors such as leg muscle strength, aerobic capacity, or strike angle as well as environmental factors like shoe mass or shoe drop might reveal other determinants of SF and DF, and might prove to be useful. Moreover, including more participants, more measures of each parameter, and a larger range of speed might increase the importance of the determinants of SF and DF, and potentially lead to additional determinants. Finally, a single running coach with 5 years of experience using the Volodalen^®^ scale has been rating the GRP of participants. Therefore, due to the fact that GRP is a key element of this study, its results would have been reinforced by having several raters. Nonetheless, the Volodalen^®^ method was shown to be reliable intra- and inter-raters (Patoz et al., 2019a).

## Practical Applications

The present study showed that age and height as well as GRP determine self-selected SF and DF, respectively, in addition to speed. However, due to the large proportion of inter-individual variations (random effects), coaches should take into account these inter-individual differences and promote self-optimization. Then, they could analyze the running technique of their runners in probably the best way (Boullosa et al., 2020).

## Conclusion

Taking into account GRP to obtain regression equations for temporal gait kinematic parameters improved the percentage of explained variance and SE only for DF, which is supported by the fact that GRP was negatively related to DF but not to SF. Therefore, these results invalidated DF regression equation proposed by Gray et al. (2019) solely based on speed but not the quadratic equation relating SF and speed. Using a broader cohort of runners, SF was shown to linearly increase with speed while a quadratic decrease was obtained for DF. However, on an individual level, SF was best described using a second-order polynomial. In addition, among age, height, BMI, weekly running distance, sex, and GRP, only age and height were shown to be positively and negatively related to SF, respectively, while none of these parameters were related to DF except GRP, which was negatively related to DF. Therefore, age and height as well as GRP are key parameters to estimate SF and DF, respectively, in addition to speed.

## Data Availability Statement

The datasets and code for this study are freely available using the access link https://github.com/aurelienPatoz/predicting-temporal-gait-kinematics.

## Ethics Statement

The studies involving human participants were reviewed and approved by the Institutional Review Board (ID RCB 2016-A00500-51). The patients/participants provided their written informed consent to participate in the original studies.

## Author Contributions

TL carried out the lab work and collected the data. TL and AP performed the data analysis and carried out the statistical analysis. AP wrote the original draft of the manuscript. AP, TL, CG, and LM critically revised the manuscript. CG and LM conceived, designed, and coordinated the study. All authors gave final approval for publication and agreed to be held accountable for the work performed therein.

## Conflict of Interest

The authors declare that the research was conducted in the absence of any commercial or financial relationships that could be construed as a potential conflict of interest.
